# Design of Boiler Welding for Improvement of Lifetime and Cost Control

**DOI:** 10.3390/ma9110891

**Published:** 2016-11-03

**Authors:** Atcharawadi Thong-On, Chatdanai Boonruang

**Affiliations:** Department of Physics and Materials Science, Faculty of Science, Chiang Mai University, Chiang Mai 50200, Thailand; mai_atcha@hotmail.com

**Keywords:** boiler, structure evolution, Fe-2.25Cr-1Mo, welding, potentiodynamic polarization, XPS (X-ray photoelectron spectroscopy)

## Abstract

Fe-2.25Cr-1Mo a widely used material for headers and steam tubes of boilers. Welding of steam tube to header is required for production of boiler. Heat affected zone of the weld can have poor mechanical properties and poor corrosion behavior leading to weld failure. The cost of material used for steam tube and header of boiler should be controlled. This study propose a new materials design for boiler welding to improve the lifetime and cost control, using tungsten inert gas (TIG) welding of Fe-2.25Cr-1Mo tube to carbon steel pipe with chromium-containing filler. The cost of production could be reduced by the use of low cost material such as carbon steel pipe for boiler header. The effect of chromium content on corrosion behavior of the weld was greater than that of the microstructure. The lifetime of the welded boiler can be increased by improvement of mechanical properties and corrosion behavior of the heat affected zone.

## 1. Introduction

Heat resistant Cr-Mo steel, Fe-2.25Cr-1Mo, has excellent high temperature strength, good creep resistance, excellent formability and low processing cost [1,2,3,4]. The good mechanical properties of the steel could be controlled by strengthening, e.g., dispersion strengthening generated by dispersion of metal carbide precipitates in the steel matrix, solid solution strengthening generated by dissolution of Mo in the steel [1,2,5], grain size strengthening [6], and so on. Steel is widely used for structures, components, and equipment in petrochemical industry and power generation industry such as thermal power plants and nuclear power plants. The components used in power generation industry, including pressure vessel wall, superheater tube, steam tube, steam pipe, and boiler header, are exposed to high humidity, high pressure (15–30 MPa), and high temperature (~550 °C) for more than 20 years [1,2,3,4,5,6,7,8]. Welding is an important heat-exposure process required for many applications of steel [2,3]. The heat-exposure process for production of boiler header includes welding, forging, and tempering [2]. A boiler rack, a bundle of steam tubes, is usually welded to a header or manifold. Because maintenance of boiler is costly and complicated, many works have emphasized the effect of weld microstructure on boiler lifetime, especially the effect on corrosion behavior, which has a great influence on the boiler lifetime, and the techniques for improvement of the boiler lifetime, e.g., use of inhibitors, control of exposure time and temperature, design of heat treatment, and design of weld repair [1,7,8,9,10,11,12]. There are three zones of the weld, fusion zone (FZ), heat affected zone (HAZ), and base metal (BM), which have different microstructures leading to different corrosion behavior [9,13]. Corrosion is an important cause of failure of the weld besides creep and fatigue decreasing strength and toughness of the weld [4,14]. Many works, using the weld materials with the same composition, have emphasized the effect of weld microstructure on corrosion behavior of boiler weld [9,10,13,15]. The results were reported in the same way that HAZ is the most susceptible part of the weld. Therefore, if the corrosion of HAZ can be controlled or the corrosion behavior is improved to be better than BM, the lifetime of the boiler weld will be improved.

A new design of materials used in welding could be introduced to improve the corrosion behavior of the weld and the cost of boiler production. In general, Fe-2.25Cr-1Mo and carbon steel are widely used for boiler production. With appropriate composition of Cr and Mo, Fe-2.25Cr-1Mo experiences better corrosion resistance and ductility [14], but at a higher cost in comparison with carbon steel. To better respond to corrosion behavior and production cost, Fe-2.25Cr-1Mo and carbon steel would be used for the steam tube and the manifold, respectively. Because the manifold is usually much thicker than the tube leading to have lower susceptibility to corrosion, the carbon steel could be used for the manifold production in order to reduce the production cost. Fe-2.25Cr-1Mo would be used for the tube, which is the smaller part with a higher susceptible to corrosion. Filler material for the welding should have a slightly higher protective influential alloying element such as Cr in comparison with the tube material, in order to increase the corrosion resistance of the critical zones such as HAZ. The aim of this work is to report on the corrosion behavior of the boiler weld with the new materials design. Correlations among corrosion behavior, chemical composition, microstructure, and hardness of the weld are discussed.

## 2. Materials and Methods

**Figure 1:**
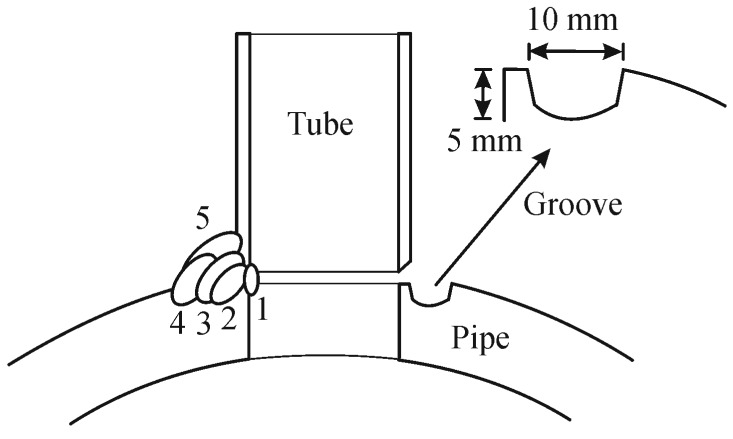
Schematic of grooved fillet joint of Fe-2.25Cr-1Mo tube and carbon steel pipe.

The composition and dimension of the commercial grade Fe-2.25Cr-1Mo tube, carbon steel pipe, and filler rod used in this study are shown in Table 1. The pipe was perforated in order to obtain a hole with 34.6 mm diameter and grooved around the hole at a distance from the hole, which could form an edge with the dimension approximately equal to the thickness of the tube. The groove dimensions were about 10 mm in width and 5 mm in depth. The tube was placed on the perforated pipe and 5 passes fillet joining, by tungsten inert gas (TIG) welding with Ar shielding gas, was performed as shown by the schematic of Figure 1. The current, voltage, travel speed, and heat input for the welding were about 150 A, 15 V, 100 mm/min, and 1.35 kJ/mm, respectively.

**Figure 2:**
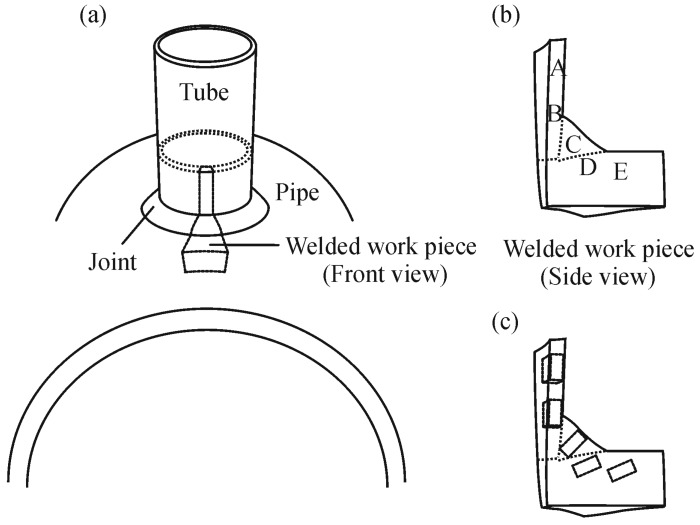
(**a**) Welded work piece; (**b**) five zones of the weld; and (**c**) samples cut for potentiodynamic polarization test.

The welded work piece was cut from the tube-pipe joint as shown in Figure 2a. The work piece was separated into five zones, indicated as A, B, C, D, and E, as shown in Figure 2b, for the zone of the tube, the zone between the tube and fusion zone, the fusion zone, the zone between the fusion zone and the pipe, and the zone of the pipe, respectively. The work piece was ground with 2000 grid SiC paper and polished with 0.3 μm alumina. The chemical composition of the five zones were analyzed using JEOL JSM-6335F scanning electron microscopy (SEM, JEOL U.S.A., Inc., Peabody, MA, USA) equipped with energy dispersive spectroscopy (EDS, JEOL U.S.A., Inc., Peabody, MA, USA) operated at 15 kV accelerating voltage. The sample was etched by 3% Nital solution for about 10 s and the microstructure was examined using optical microscopy. Vickers microhardness test for each zone was performed with 0.98 N load and 10 s loading time. Corrosion behavior was studied using potentiodynamic polarization test. Each of the five zones of the work piece shown in Figure 2b was cut into a sample with dimensions of about 2.5 × 6.5 × 2.0 mm as shown in Figure 2c.

Each sample was connected to an electric wire by silver paint and was mounted in epoxy resin in order to obtain exposure surface area of about 0.16 cm^2^. The exposure surface was ground with 2000 grid SiC paper. Potentiodynamic polarization test was performed in 3.5% NaCl solution with Ag/AgCl reference electrode and Pt counter electrode. The test potential and the potentiodynamic polarization curve were controlled and generated by Metrohm FRA2 µAUTOLAB TYPE III potentiostat cooperated with NOVA software (version 1.10, Metrohm, Herisau, Switzerland). The polarization was run from about −200 to 200 mV related to an open circuit potential (OCP) with the rate of 1 mV/s. The curve was analyzed using Tafel extrapolation method in order to evaluate potentiodynamic polarization parameters. The equivalent weight, *W_E_*, and the corrosion rate, *R_C_* (mm/year), were calculated using Equations (1) and (2) [16,17,18]:

(1) $$W_{E}=\frac{1}{\sum \frac{n_{i}f_{i}}{w_{i}}},$$

where *n_i_*, *f_i_*, and *w_i_* are valence, weight fraction, and equivalent atomic weight of the *i*^th^ element of the test alloy, respectively. *K* is the constant (3.27 × 10^–3^ mm·g/(µA·cm·year)), *i_corr_* is a corrosion current density (µA/cm^2^), and *ρ* is a density of the alloy (g/cm^3^). After the polarization test, a sample surface was ground with a 2000 grid SiC paper, dried by blown air, and brought to immersion test within 10 min. The immersion test was done in 3.5% NaCl solution for 1 h. The samples were cleaned with distilled water and dried by air blow. The samples were examined using Kratos Axis ULTRA^DLD^ X-ray photoelectron spectroscopy (XPS, Kratos Analytical Ltd., Manchester, UK) equipped with a monochromatic source of Al Kα X-ray. The base pressure in the analysis chamber was about 5 × 10^−9^ torr. The X-ray source was operated with a spot size of 700 × 300 µm at 150 W; 15 kV and 10 mA; with initial photo energy of 1.4 keV. The binding energy of the adventitious C 1s peak at 285 eV was used for calibration of wavelength shift. The spectra were recorded and analyzed, with the pass energy and energy step of 20 and 0.1 eV, respectively, using VISION II software (version 2.2.9, Kratos Analytical Ltd., Manchester, UK). Surface morphology of the samples was examined using SEM operated at 15 kV accelerating voltage.

## 3. Results and Discussion

### 3.1. Composition and Structure of the Weld

Main compositions, in wt.%, of the five zones of the weld analyzed by EDS are shown in Table 2. The compositions exhibited results corresponding to those of the original materials shown in Table 1. Zone D, which was the boundary between Zones C and E, exhibited diffusion of Cr and Mo from Zone C. Cr:Fe ratio indicated quantity of Cr which could lead to increase in corrosion resistance of any zone by increase in formation of chromium oxide. Improvement of corrosion resistance of low alloy steels by adding of Cr was reported in literature in which low alloy steels had better corrosion behavior in comparison with carbon steels [18].

**Figure 3:**
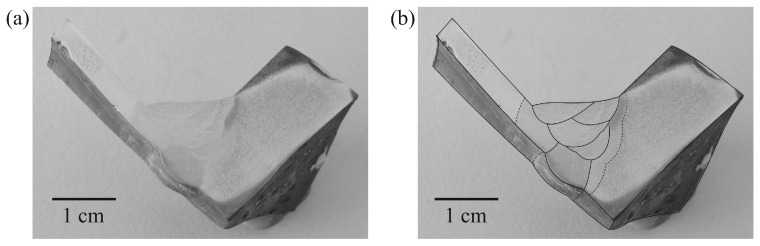

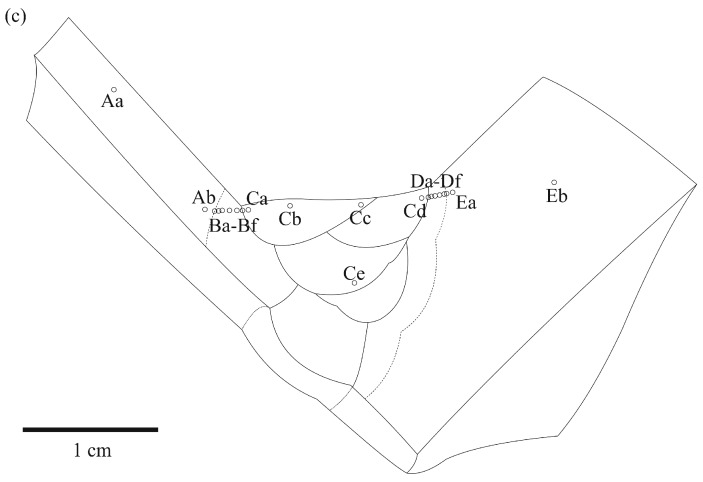
(**a**) Macrostructure; (**b**) schematic; and (**c**) examined positions of the weld.

**Figure 4:**
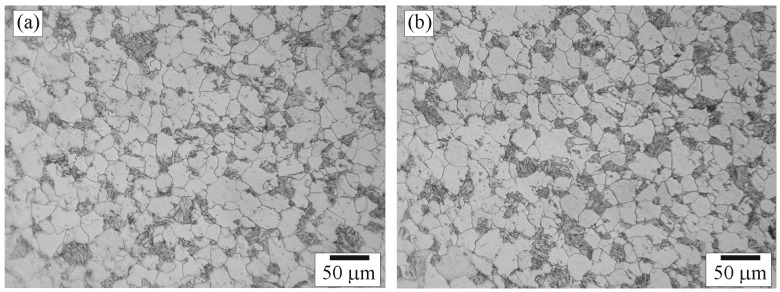
Optical micrographs of positions: (**a**) Aa; and (**b**) Ab in Zone A.

After etching, macrostructure of the weld appeared as shown in Figure 3a. The schematic of the weld is shown in Figure 3b. Microstructure of the weld was examined across the five zones by an optical microscopy and the examined positions were indicated as shown in Figure 3c. Microstructures of Zone A selected for examination were those of positions Aa, in BM far away from A–B zone interface, and Ab, close to A–B zone interface. The microstructures of positions Aa and Ab are shown in Figure 4a,b, respectively. The micrographs show that positions Aa and Ab had the same microstructure composed of ferrite grains and pearlite colonies with the size of about 20–40 µm. It is suggested that the temperature exposure to position Ab was not high enough for the change of microstructure so that position Ab was regarded as in BM as well.

**Figure 5:**
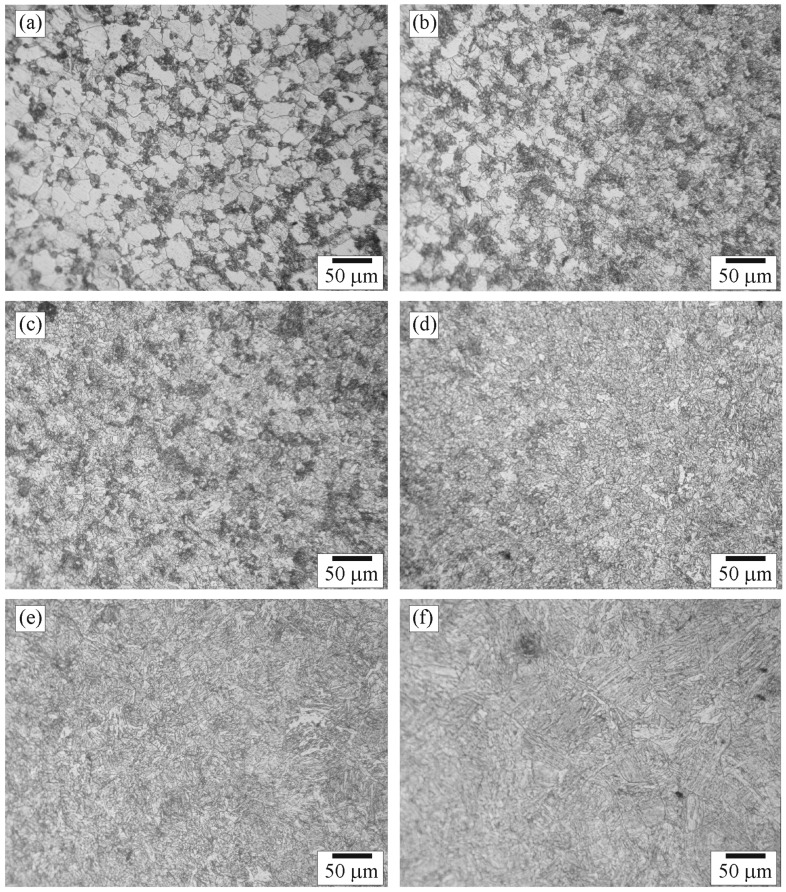
Optical micrographs of positions: (**a**) Ba; (**b**) Bb; (**c**) Bc; (**d**) Bd; (**e**) Be; and (**f**) Bf in Zone B.

Microstructures of Zone B selected to be examined were those of positions Ba (close to the A–B zone interface), Bf (close to the B–C zone interface), and Bb–Be (between positions Ba and Bf). The microstructures of positions Ba–Bf are shown in Figure 5a–f. Figure 5a shows that pearlite colonies were affected by heat and recrystallized while ferrite grains were not recrystallized. In general, the grain recrystallization is associated with energy storage in structure. A large-stored-energy structure would exhibit early recrystallization. Therefore, the pearlite colonies storing larger energy were recrystallized earlier than the ferrite grains [19]. The size of recrystallized-pearlite colonies was about 1–5 µm. Figure 5b shows the change of microstructure from unaffected ferrite to recrystallized ferrite in position Bb. It is believed that the temperature exposure to the right of position Bb was high enough to cause recrystallization of the ferrite. The size of the recrystallized-ferrite grains was about 5–15 µm. Figure 5c shows that all the pearlite and ferrite in position Bc were recrystallized. Figure 5d shows the change of microstructure from recrystallized pearlite and ferrite to bainite in position Bd. It is obvious that the quantity of pearlite colonies (dark) and ferrite grains (light) decreased and that of coarse-bainite colonies (medium) increased as the distance from the left to the right increased. It is suggested that the temperature exposure to the right of position Bd was high enough and the cooling rate was appropriate for the formation of bainite. Figure 5e shows that the microstructure of position Be was completely coarse bainite with a grain size of about 20–60 µm. The grain size tended to increase as the distance from left to right increased, reflecting that grain growth occurred in this region. Figure 5f shows the change of microstructure from equiaxed bainite colonies, located on the left, to elongated bainite colonies, diagonally oriented and located on the top-right corner of the micrograph. In general, an orientation of bainite correlates with that of austenite because bainite occurred by transformation of austenite. The correlation of crystallographic direction and plane between bainite and austenite can be expressed as <111>_B_//<110>_A_ and {1-10}_B_//{1-11}_A_ [20], where B and A denote parameters of bainite and austenite, respectively. Therefore, after transformation of austenite during welding, bainite presented in the weld had preferred orientation of <111>{1-10}.

**Figure 6:**
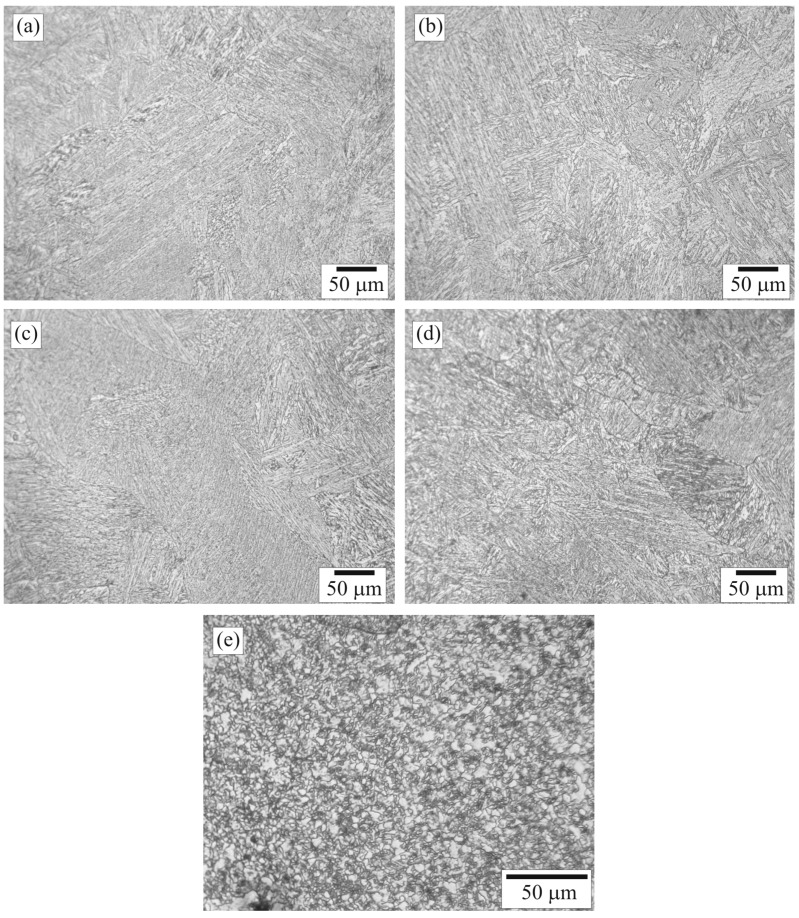
Optical micrographs of positions: (**a**) Ca; (**b**) Cb; (**c**) Cc; (**d**) Cd; and (**e**) Ce in Zone C.

Microstructures of Zone C selected for examination were those of positions Ca (close to B–C zone interface), Cc (close to the 4th pass joint), Cb (etween positions Ca and Cc), Cd (close to C–D zone interface), and Ce (in the middle of Zone C). The microstructures of positions Ca–Ce are shown in Figure 6a–e. Figure 6a shows that the microstructure of position Ca was elongated bainite colonies oriented diagonally from the bottom-left corner to the top-right corner of the micrograph. The microstructure of position Cb is shown in Figure 6b. The bainite colonies were oriented in vertical. The microstructures of positions Cc and Cd are shown in Figure 6c,d, respectively. The microstructures were elongated bainite colonies as well. The colonies were oriented diagonally from the bottom-right corner to the top-left corner of the micrographs. It is believed that the temperature and cooling rate in this region were suitable for formation of bainite. In addition, Cr and Mo contents of the weld materials could promote bainite formation [10]. Formation of bainite in FZ, without formation of acicular ferrite, reflected no inclusion presented in FZ caused by no inclusion forming elements such as Al or Ti in the weld materials [21,22]. The elongated grain generated during solidification of the molten filler in FZ could be an epitaxial growth; grain growth, in particular crystallographic direction, paralleled to the orientation of crystal in contact with the melt [22]. The grain orientation of any position in FZ was depended on the growth direction from the cool interface, the former pass joint. The grain size of position Cd was quite smaller than that of the other positions reflecting the high cooling rate caused by large quantity of heat conducted through the pipe, the C–D zone interface. The microstructure of position Ce is shown in Figure 6e. Effect of tempering by latter pass joint on microstructure of former pass joint has been revealed. In general, heat generated from latter pass can effect on microstructure of former pass joint. A number of different structures, recrystallization, and grain growth can be generated in the former pass region as reported in the literature [12]. In this study, granular bainite was presented in the former pass region. After being tempered, formerly bainite could transform into a structure with granular bainites, which could merge together and become coarser. The microstructure is consistent with the literature [23].

**Figure 7:**
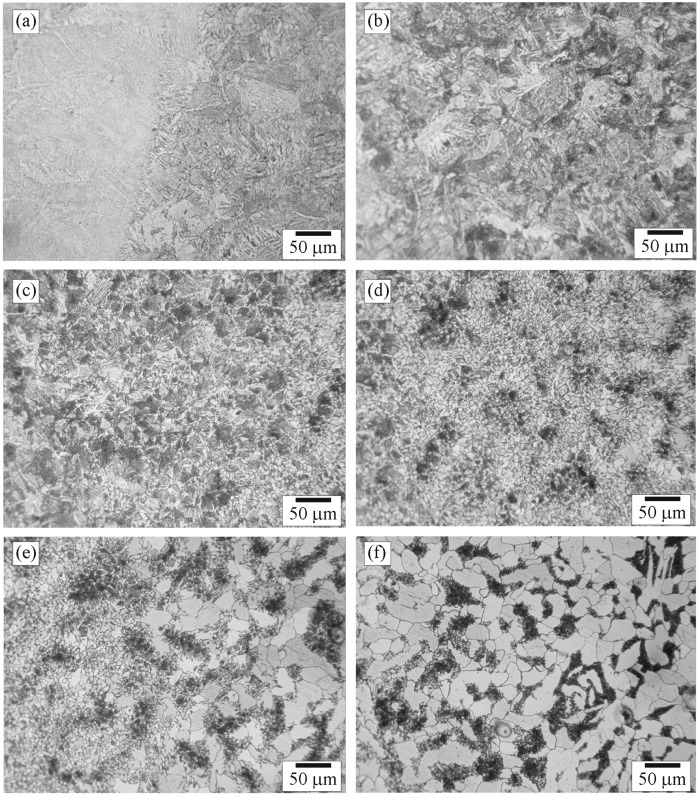
Optical micrographs of positions: (**a**) Da; (**b**) Db; (**c**) Dc; (**d**) Dd; (**e**) De; and (**f**) Df in Zone D.

Microstructures of Zone D selected for examination were those of positions Da (close to the C–D zone interface), Df (close to the D–E zone interface), and Db–De (between positions Da and Df). The microstructures of positions Da–Df are shown in Figure 7a–f. Figure 7a shows the change of microstructure from bainite colonies, located on the left, to a mixed structure of fine-pearlite colonies (dark), ferrite grains (light), and coarse-bainite colonies (medium) located on the right of the micrograph. According to dimension of the pipe was larger than that of the tube, cooling rate of the pipe would be lower than that of the tube leading the microstructure of the pipe shown in Figure 7a was different from that of the tube shown in Figure 5f. Figure 7b shows the microstructure of position Db composed of coarse bainite, fine pearlite, and ferrite with grain sizes of 20–60, 10–35, and 5–30 μm, respectively.

Figure 7c shows the microstructure of position Dc composed of coarse bainite, fine pearlite, and ferrite with grain sizes of 15–40, 5–35, and 5–20 μm, respectively. The grain size in Figure 7c is approximately smaller than that in Figure 7b and larger than that in Figure 7d, reflecting the grain growth occurred in positions Db and Dc. Figure 7d shows the microstructure of position Dd composed of recrystallized pearlite colonies and ferrite grains with grain sizes of 5–20 and 3–15 μm, respectively. No bainite was observed in this region. It is believed that temperature and cooling rate in this region were not appropriate for formation of bainite. Figure 7e shows the microstructure of position De composed of recrystallized pearlite colonies, recrystallized ferrite grains, and unaffected ferrite grains with grain sizes of 5–15, 5–15, and 20–30 μm, respectively. It is obvious that there was a change of ferrite from unaffected ferrite, located on the right, to recrystallized ferrite, located on the left of the micrograph. It is believed that the temperature exposure to the left of position De was high enough to allow recrystallization of ferrite. Figure 7f shows that pearlite colonies were affected by heat and were recrystallized while ferrite grains were not recrystallized as shown in Figure 5a. The recrystallization increased moving from right to left in the micrograph, in other words, as the exposure temperature increased.

**Figure 8:**
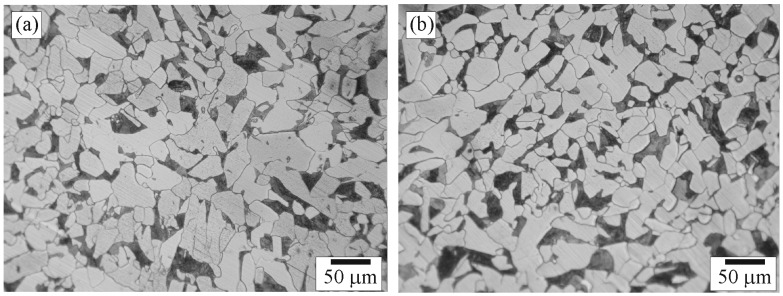
Optical micrographs of positions: (**a**) Ea; and (**b**) Eb in Zone E.

Microstructures of Zone E selected for examination were those of positions Ea (close to D–E zone interface), and Eb (in BM far away from D–E zone interface). The microstructures of positions Ea and Eb are shown in Figure 8a,b, respectively. The micrographs show that positions Ea and Eb had the same microstructure composed of unaffected-ferrite grains and unaffected-pearlite colonies with sizes of about 10–70 and 5–35 µm, respectively. It is suggested that the temperature exposure to position Ea was not high enough for recrystallization leading it to be regarded as in BM.

The weld microstructure was affected by heat generation and extraction. The different exposure temperatures and cooling rates during welding of any zones could lead them to have different microstructures. The weld microstructure was consistent with many works reported on weld microstructure of steels [4,9,10,12,22,24,25,26].

### 3.2. Hardness of the Weld

**Figure 9:**
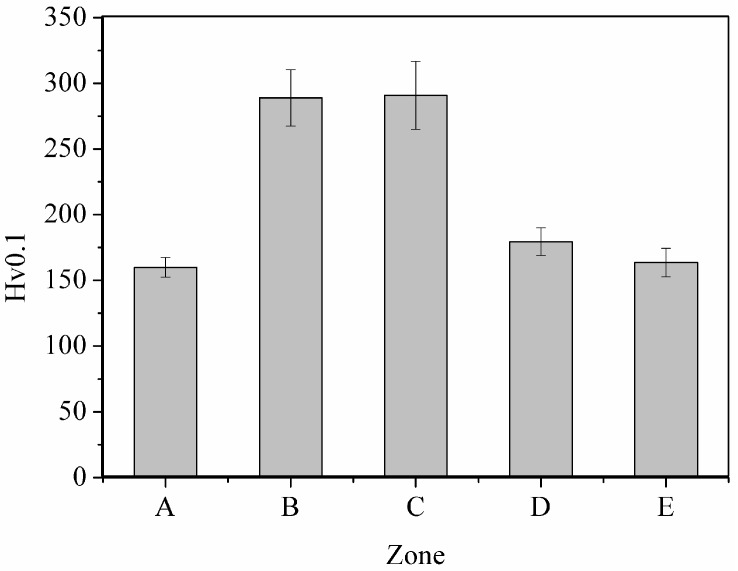
Hardness of the five zones of the weld.

Mechanical properties of the weld were characterized by microhardness tests performed on the five zones and the results are shown in Figure 9. Hardnesses of Zones A and E were 160.0 ± 7.4 and 163.6 ± 10.9 HV, respectively, and were low in comparison with those of other zones. The structure of pearlite and ferrite in the BMs, as shown in Figure 4 and Figure 8, possessed low hardness and low strength leading the BMs to experience low hardness. Hardness of Zone D, 179.4 ± 10.5 HV, was higher than that of Zone E. Zone D, shown in Figure 7, is composed of various structures: unaffected ferrite, recrystallized pearlite, recrystallized ferrite, growth pearlite, growth ferrite, and bainite. Therefore, the hardness of Zone D was influenced by hardness of these structures. Even though Zone B was regarded as HAZ, the same as Zone D, hardness of Zone B, 288.9 ± 21.6 HV, was obviously higher than that of Zone D. Figure 5 and Figure 7 show that even though the microstructure type was the same in Zones B and D, the quantity of bainite in Zone B was higher than in Zone D, especially in the region near the B–C zone interface which was entirely bainite. In general, hardness of the weld increases as the bainite content increases [15,24]. Therefore, it is obvious that bainite played an important role in promoting hardness in any zones. Hardness of Zone C, 290.8 ± 25.9 HV, was approximately the same as that of Zone B. As shown in Figure 6, even though the majority microstructure of Zone C was bainite with granular bainite presented in some regions, the bainite colonies were large-elongated grains. Dislocation can glide in longer distance in large grain causing hardness and strength to be lower than small grain. The total effect of structure and grain size caused no significant difference in hardness between Zone C (composed of large elongated bainite colonies and granular bainite) and Zone B (composed of small equiaxed bainite colonies, recrystallized pearlite, recrystallized ferrite, and unaffected ferrite grains).

### 3.3. Corrosion Behavior

**Figure 10:**
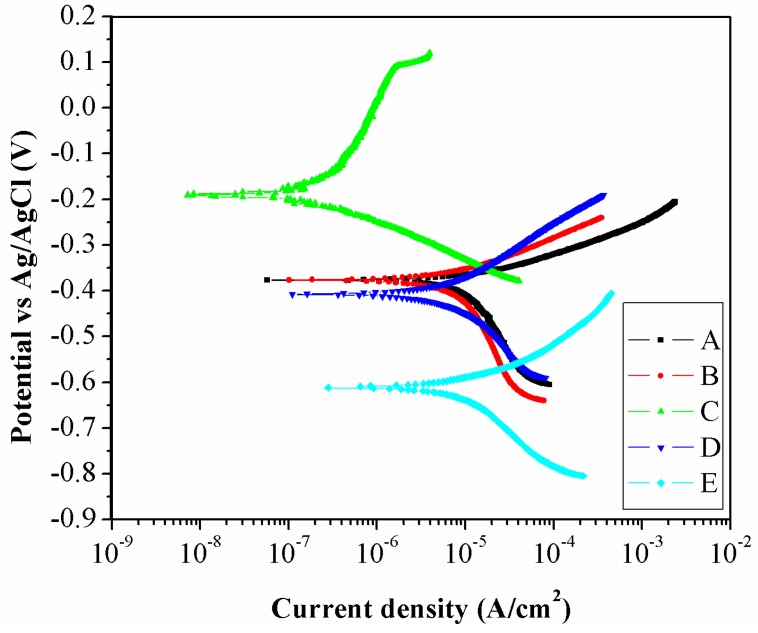
Potentiodynamic polarization curves of the five zones of the weld.

Potentiodynamic polarization curves of the five zones of the weld are shown in Figure 10. All specimens exhibited only active behavior since no passive behavior is shown in the anodic curves. The parameters obtained from curve analysis and calculated corrosion rates of any zones of the weld (using the chemical composition in Table 2) are shown in Table 3. The *E_corr_* and *i_corr_* of Zone E were the lowest and highest, respectively, in comparison with other zones, reflecting highest tendency to be corroded. The calculated corrosion rate of Zone E was the highest at 0.1353 mm/year. The *E_corr_* and *i_corr_* of Zone A were higher and lower than those of Zone E, respectively, reflecting lower corrosion susceptibility caused by effect of higher Cr content. The *E_corr_* and *i_corr_* of Zone C were the highest and lowest, respectively, in comparison with other zones, indicating lowest corrosion susceptibility. The corrosion rate of Zone C was the lowest at 0.0024 mm/year. The *E_corr_* and *i_corr_* of Zones D and B were higher and lower than Zones E and A, respectively, reflecting lower corrosion susceptibility.

In general, when composition of an influential alloying element such as Cr of work piece and filler are about the same, the corrosion susceptibility of the weld would depend on the microstructure. High energy structure generally occurred by non-equilibrium transformation and to be regarded as non-equilibrium structure having low thermodynamically stability. Effects of heat and cooling rate on HAZ could promote generation of high energy structure such as bainite resulting in high corrosion susceptibility in comparison with BM [24]. Furthermore, mixed microstructure of bainite and ferrite, having different internal energy, establishes local anode and local cathode in HAZ leading to have high corrosion susceptibility in comparison with ferrite in BM [15]. However, the weld in our study exhibited different results. Even though mixed microstructure was generated in the weld, compositions of Cr in work pieces and filler were significantly different. In this study, it is obvious that the Cr content had greater effect, on corrosion behavior, than the microstructure since HAZ, with higher Cr content, had better corrosion behavior in comparison with BM. The explanation is consistent with the effect of composition reported in literature [27]. It is suggested that a corrosion product of chromium rich oxide played an important role to improve corrosion resistance of HAZ leading to decrease in tendency of weld failure.

### 3.4. X-ray Photoelectron Spectroscopy

**Figure 11:**
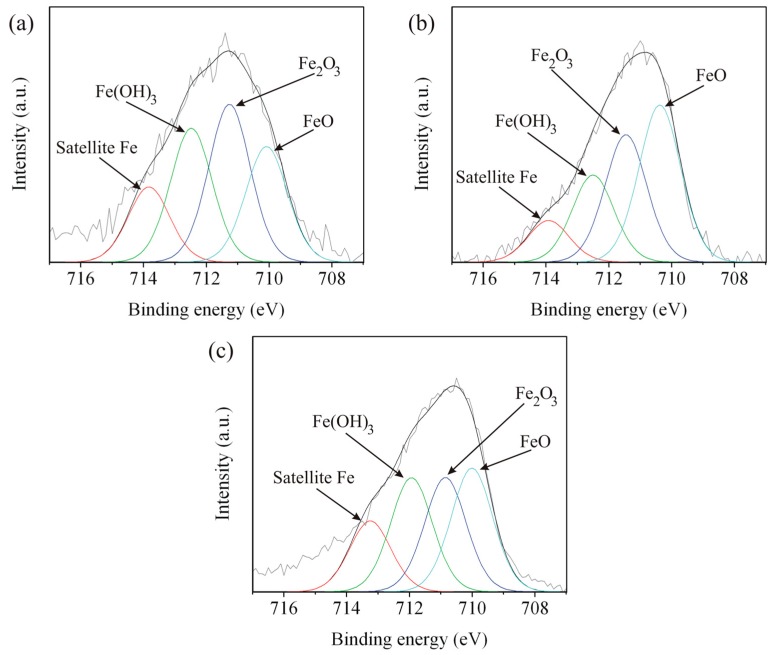
Fe 2p spectra of Zones: (**a**) A; (**b**) C; and (**c**) E.

**Figure 12:**
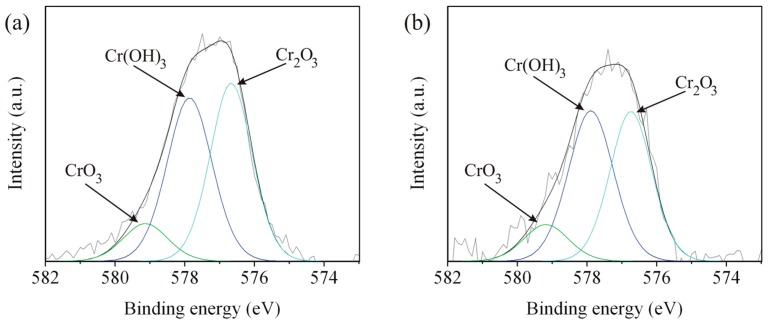

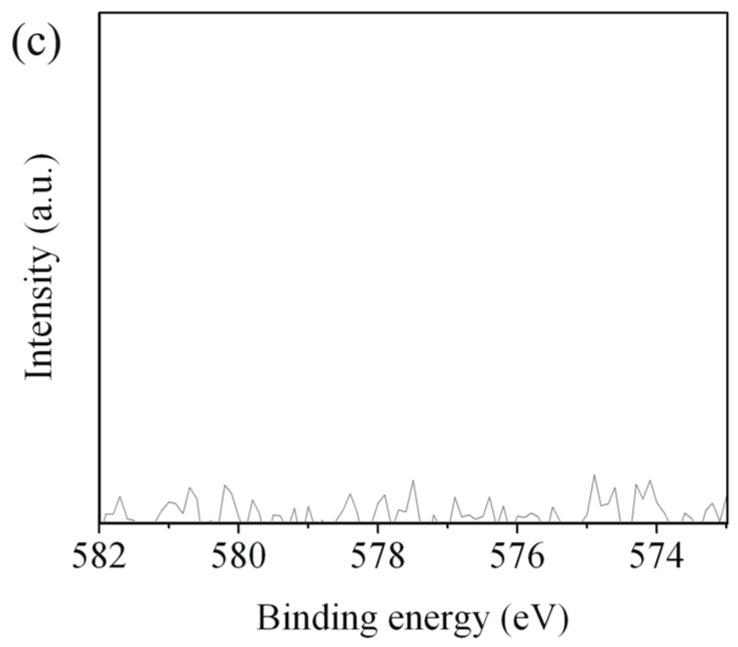
Cr 2p spectra of Zones: (**a**) A; (**b**) C; and (**c**) E.

**Figure 13:**
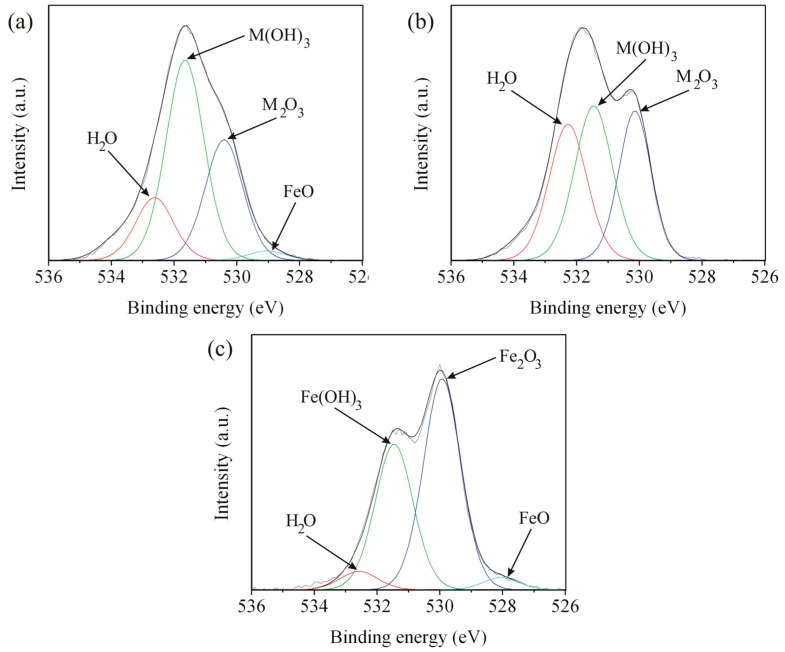
O 1s spectra of Zones: (**a**) A; (**b**) C; and (**c**) E.

Surface characterization by XPS after immersion test, for the five zones of the weld, was done for better clarification of corrosion behavior of the weld. The Fe 2p, Cr 2p, and O 1s spectra of Zones A, C, and E are shown in Figure 11, Figure 12 and Figure 13, respectively. The spectra of Zones B and D seemed to have the same characteristic as Zones A and E, respectively, and will not be presented here. The deconvoluted peaks of FeO, Fe_2_O_3_, Fe(OH)_3_, and satellite Fe are presented at binding energies of about 710.16, 711.19, 712.31, and 713.67 eV, respectively. The results correspond to the reported literature values [28,29]. It is obvious that FeO and Fe_2_O_3_ were formed on Zones A, C, and E. Fe_3_O_4_ could possibly form as well since the iron in Fe_3_O_4_ has 2 oxidation states or the compound structure was a combination of FeO and Fe_2_O_3_. Detection of Fe(OH)_3_ indicated high humidity of the weld after the immersion test. The deconvoluted peaks of Cr_2_O_3_, Cr(OH)_3_, and CrO_3_ are present in the spectra of Zones A and C at binding energies of about 576.70, 577.86, and 579.14 eV, respectively, and the results correspond to literatures [28,29,30,31,32]. There is no Cr 2p spectrum since there was very small quantity of chromium in Zone E as mentioned in Table 2. There were two oxidation states of Cr for the chromium oxides formed in Zones A and C, reflecting a different level of oxygen in the weld. The formation of Cr(OH)_3_ is believed to be followed the same mechanism as that of Fe(OH)_3_. The peak of FeO appears in the O 1s spectra of Zones A and E at a binding energy of about 528.55 eV. The peaks of oxide, hydroxide, and water appear in the spectra of Zones A, C, and E at binding energies of about 530.18, 531.54, and 532.51 eV, respectively, and the results correspond to the literature [28,29,33]. M in M_2_O_3_ and M(OH)_3_ in Figure 13 represent Fe or Cr since peaks of oxides and hydroxides of iron and chromium were detected in Fe 2p and Cr 2p spectra of Zones A and C. According to the important parameters such as *E_corr_* and *i_corr_* shown in Table 3 and the XPS results shown in Figure 11, Figure 12 and Figure 13, it is evident that Cr content in weld materials had an influence on formation of Cr_2_O_3_ affecting corrosion behavior of any zones of the weld. The maximum and minimum Cr content zones such as Zones C and E, respectively, would be a good comparative condition to indicate the effect of Cr content on corrosion behavior of the weld. With low Cr content, Cr_2_O_3_ could not be formed or detected in Zone E leading to exhibit poor corrosion behavior (experience low *E_corr_* and high *i_corr_*). As for Zone C, Cr_2_O_3_ could be formed leading to exhibit better corrosion behavior (experience higher *E_corr_* and lower *i_corr_*).

### 3.5. Scanning Electron Microscopy

**Figure 14:**
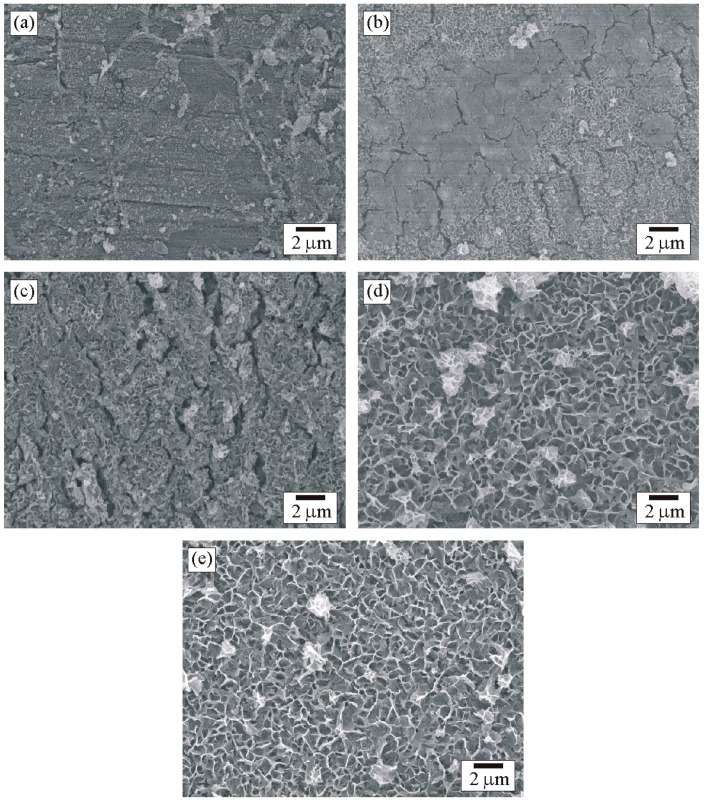
SEM micrograph of the surface of the five zones of the weld after the immersion test: (**a**) Zone A; (**b**) Zone B; (**c**) Zone C; (**d**) Zone D; and (**e**) Zone E.

After the immersed samples were characterized by XPS, unfortunately, they had to be kept in desiccator for more than 24 h before characterized by SEM, resulting in dehydration of hydroxide and formation of oxide. SEM micrograph in Figure 14 reveal surface morphology of the five zones of the weld. Various morphologies of oxides were found on some areas of the surface of each zone. A major morphology of each zone was selected to be a representative of each zone. Small grains with fine particles of oxides were presented in Zones A and B. The oxides presented in Zone C exhibited the same morphology but more abundant in comparison with Zones A and B. Morphology of the oxide corresponded to Cr_2_O_3_ in the literature [34], reflecting the formation of Cr_2_O_3_ in these zones and resulting in good corrosion behavior. The results correspond to those of XPS and potentiodynamic polarization. It is obvious that morphology of oxide in Zones D and E was different in comparison with Zones A, B, and C. A porous-honeycomb oxide was presented abundantly in Zones D and E. The morphology corresponded to Fe_3_O_4_ in the literature [10], indicating rich formation of Fe_3_O_4_ in these zones. Without the formation of chromium oxide, corrosion behavior of these zones would be poor. Chemical composition of the weld materials played an important role compared to microstructure in formation of oxide and corrosion behavior of the weld.

## 4. Conclusions

The welding temperature and chemical composition of the weld had an influence on the weld microstructure and oxide formation. The effects of chromium content and formation of Cr_2_O_3_ on corrosion behavior of the weld were greater than that of the microstructure. The microstructure had an influence on the hardness of the weld. Formation of bainite had a great influence on hardness increase of the weld. New materials design for boiler welding to improve the lifetime and cost control using TIG welding of Fe-2.25Cr-1Mo tube to carbon steel pipe with chromium-containing filler is completed. The cost of production could be reduced by the use of carbon steel pipe instead of Fe-2.25Cr-1Mo pipe for boiler header. The HAZ experienced better mechanical properties and corrosion behavior in comparison with BM, which could lead to increase in lifetime of the welded boiler.

## Figures and Tables

**Table 1:** Chemical composition and dimension of tube, pipe, and filler rod used in this study.

Materials	Composition (wt.%)								Dimension (mm)	
	C	Si	Mn	P	S	Cr	Mo	Fe	OD ^1^	ID ^2^
Tube	0.10	0.27	0.45	0.12	0.04	2.12	0.96	Bal.	38.1	34.6
Pipe	0.15	0.49	0.61	0.00	0.00	0.07	0.22	Bal.	219.1	207.1
Filler rod	0.08	0.56	0.66	0.01	0.00	2.42	0.97	Bal.	2.4	-

^1^ Outer diameter; ^2^ Inner diameter.

**Table 2:** Chemical composition of the five zones of the weld analyzed by EDS.

Zone	Composition (wt.%)			Cr:Fe
	Fe	Cr	Mo	
A	97.01	1.77	1.22	0.0182
B	96.95	1.90	1.15	0.0196
C	96.11	2.43	1.46	0.0253
D	97.17	2.01	0.82	0.0207
E	99.99	0.01	0.00	0.0001

**Table 3:** Potentiodynamic polarization parameters of the five zones of the weld.

Zone	*β_a_* (V/dec)	*β_c_* (V/dec)	*E_corr_* (V)	*i_corr_* (µA/cm²)	*R_C_* (mm/Year)
A	0.0666	−0.2406	−0.389	9.35	0.1069
B	0.0806	−0.2654	−0.377	7.31	0.0835
C	0.2741	−0.0828	−0.192	0.22	0.0024
D	0.1182	−0.2212	−0.393	7.44	0.0851
E	0.1091	−0.1861	−0.627	11.66	0.1353
